# Weathering the storm of emotions: immediate and lasting effects of reinterpretation and distancing on event-related potentials and their association with habitual use of cognitive reappraisal

**DOI:** 10.3758/s13415-023-01105-4

**Published:** 2023-05-25

**Authors:** Raphaela I. Zehtner, Marie K. Neudert, Axel Schäfer, Susanne Fricke, Rosa J. Seinsche, Rudolf Stark, Andrea Hermann

**Affiliations:** 1 Department of Psychotherapy and Systems Neuroscience, Justus Liebig University Giessen, Otto-Behaghel-Str. 10H, Giessen, 35394 Germany; 2 Bender Institute of Neuroimaging, Justus Liebig University Giessen, Gießen, Germany; 3 Center for Mind, Brain and Behavior (CMBB), University of Marburg and Justus Liebig University Giessen, Marburg, Giessen, Germany

**Keywords:** Emotion regulation, Cognitive control, EEG, LPP, P300, Negative feelings, Neural

## Abstract

Reinterpretation and distancing, two cognitive reappraisal tactics, are known to effectively reduce negative feelings and event-related potentials (ERPs), such as the P300 and the late positive potential (LPP), in the short-term. Less is known about differential and lasting effects on ERPs as well as their association with habitual reappraisal. Fifty-seven participants were instructed to passively view or reappraise (reinterpretation, distancing) pictures that were repeatedly presented with the same instruction (active regulation phase). Thirty minutes later, these pictures were shown again without instruction for the assessment of lasting effects (re-exposure phase). ERPs were recorded and participants rated the intensity of negative feelings following picture presentation. Reappraisal led to an attenuation of the LPP, and both tactics decreased negative feelings during active regulation, whereby reinterpretation had a stronger impact on the subjective level. Passive re-exposure resulted in reduced negative feelings for previously reappraised pictures but had no lasting effects on ERPs. Higher habitual reappraisal was associated with higher P300 and early LPP amplitudes for emotional reactivity during the active regulation phase. During the re-exposure phase, higher habitual reappraisal was not related to ERPs. The current findings emphasize the effectiveness of both tactics in the short-term and lasting effects on the subjective experience of negative feelings. Enhanced emotional reactivity on the electrocortical level in individuals with a more frequent habitual use of reappraisal might indicate a higher preparedness to regulate.

## Introduction

An adaptive and flexible use of emotion regulation strategies is crucial to well-being and social functioning in healthy (Brockman et al., 2017; Gross and John, 2003) as well as clinical populations (Berking et al., 2008; Kraiss et al., 2020; Sloan et al., 2017). Thereby, emotion regulation is a core element of transdiagnostic treatments of mental disorders (Sloan et al., 2017). Fears and cognitive distortions often are addressed by cognitive restructuring via cognitive reappraisal (Beck, 1976; Klumpp et al., 2014), which refers to cognitively construing a potentially emotion-eliciting situation in a way that alters its emotional impact (Gross and John, 2003). In this context, trainability and enduring effects of cognitive reappraisal as well as the association with habitual use of this strategy are of great importance.

For the immediate effect of emotion regulation during active regulation, a large body of research has shown cognitive reappraisal to be effective in reducing the experience of negative feelings (Webb et al., 2012) and in the modulation of neural response patterns to emotional stimuli as shown via electroencephalography (EEG) (for reviews see, Dennis and Hajcak, 2009; Hajcak and Foti, 2020; Hajcak et al., 2010; Olofsson et al., 2008). In the EEG, the P300 and late positive potential (LPP) have been demonstrated to be sensitive to arousal-related changes by cognitive reappraisal, leading to reduced amplitudes compared with passively watching aversive pictures (Hajcak and Foti, 2020; Hajcak et al., 2010; Hajcak and Nieuwenhuis, 2006; Krompinger et al., 2008; Qi et al., 2017; Thiruchselvam et al., 2011; Willroth and Hilimire, 2016). The P300 (being maximal approximately 300–600 ms after stimulus onset) indexes stimulus significance for motivationally salient stimuli (Hajcak and Foti, 2020; MacNamara et al., 2022), both being nonemotional (e.g., detecting targets; Gonsalvez et al., 2007) and emotional in nature (e.g., aversive compared with neutral pictures, often referred to as emotional reactivity; Hajcak et al., 2010; Weinberg et al., 2012). The LPP might resemble a sustained version of the P300, beginning approximately 400 ms after stimulus onset and lasting for several seconds, whereby it is associated with emotional meaning (Hajcak et al., 2010; MacNamara et al., 2022). Different reappraisal tactics, such as distancing and reinterpretation, have not always been examined distinctly (Cao et al., 2020; Moser et al., 2009; Moser et al., 2010; Thiruchselvam et al., 2011), although there seems to be a different pattern in timing and effectiveness (Qi et al., 2017; Willroth and Hilimire, 2016). While reinterpretation implies altering the meaning of a stimulus or situation (e.g., imagining a better ending of the depicted situation), distancing is defined as changing one’s personal connection to or psychological distance from a stimulus or situation (e.g., taking the perspective of a detached observer) (Ochsner et al., 2012). Previous studies on event-related potentials (ERPs) show inconsistent results, with only reinterpretation (also compared with distancing) (Willroth and Hilimire, 2016) or both tactics (Qi et al., 2017) to reduce the LPP during active regulation.

Lasting effects of cognitive reappraisal on ERPs during passive re-exposure to previously reappraised pictures have only been investigated in a few studies. On a self-report level, reappraisal-related modulations are reported to last for shorter periods (such as 30 minutes; Qi et al., 2017; Thiruchselvam et al., 2011), over 1 day (Hermann et al., 2017) up to 1 week (Ahn et al., 2015 for men only; Denny and Ochsner, 2014; Hermann et al., 2021). For the EEG, Thiruchselvam et al. (2011) showed a lasting effect on LPP reduction of previously reappraised pictures (compared with previously passive watching) during re-exposure 30 minutes later. So far, only one ERP study differentiated between lasting effects of reinterpretation and distancing (Qi et al., 2017), indicating a reduced centroparietal LPP amplitude during passive re-exposure 30 minutes later, specifically for pictures previously distanced from (but not for reinterpretation). They further demonstrated a lasting effect of reinterpretation on the reported valence of the pictures and of distancing on arousal ratings (Qi et al., 2017).

In this context, individual differences in the habitual use of reappraisal have not been accounted for, which might impact the effectiveness of both tactics. Previous research has demonstrated habitual use of reappraisal to be associated with stronger reductions of the LPP amplitudes during both, passively watching aversive stimuli (Harrison and Chassy, 2019), as well as the implementation of reinterpretation (Moser et al., 2014). However, no studies to date examined the association of habitual use of cognitive reappraisal with emotional reactivity and immediate or lasting effects of the different reappraisal tactics reinterpretation and distancing.

Therefore, the goals of our study were to examine the association of habitual reappraisal with spontaneous responding to aversive compared to neutral pictures, i.e., emotional reactivity, as well as with immediate (active emotion regulation phase) and lasting effects (30 minutes later, re-exposure phase) of reinterpretation and distancing. We hypothesized that looking at aversive pictures (compared with neutral ones) should lead to a stronger experience of negative feelings and higher LPP amplitudes, whereby a greater habitual use of reappraisal should be related to less emotional reactivity during active regulation and re-exposure. Furthermore, we assumed that the implementation of both tactics should result in a decrease of negative feelings and LPP amplitudes (compared with passively looking at aversive pictures) during active regulation as well as during re-exposure, which also should be associated with a greater use of habitual cognitive reappraisal.

## Methods and materials

### Participants

This study is part of a larger project investigating emotion regulation in healthy participants as well as patients with emotional disorders. Seventy-six healthy participants volunteered in the current study, recruited via mailing lists and a participant database at the local university, as well as via public notice boards. A short telephone interview was conducted to screen for inclusion and exclusion criteria of the study and to set two appointments. On the first appointment, participants were screened with the Diagnostic Interview for mental disorders for DSM-5 (DIPS; Margraf, Cwik, Pflug, and Scheider, 2017a; Margraf, Cwik, Suppiger, and Schneider, 2017b) to ensure that participants did not meet the full criteria of any mental disorder currently as well as in the past. They further received questionnaires to complete until the next appointment on which the emotion regulation paradigm was conducted. The following criteria led to exclusion from the study: mental disorders (current or past), chronic or severe medical diseases or neurological disorders, psychotropic drug intake, psychological treatment (current or past), scalp injuries, pregnancy, and left-handedness as assessed by the Edinburgh Inventory of Handedness (Oldfield, 1971). Participants had to be aged between 18 and 65 years, had normal or corrected-to-normal vision, and spoke German fluently. All participants were reimbursed with course credits or 8€/h and provided written, informed consent according to the guidelines of the ethical standards of the Declaration of Helsinki. The study was approved by the local ethical review board of the Faculty of Psychology and Sports Science at the Justus Liebig University Giessen, Germany.

In all, 19 screened participants had to be excluded. The reasons were as following: not appearing to the second appointment due to the pandemic condition or due to private reasons (*n* = 5), meeting criteria for a mental disorder (*n* = 6) or having received psychotherapy in the past (*n* = 2), technical problems during EEG recordings (*n* = 2), and excessive artifacts in the EEG (*n* = 2). Inspection of the reappraisal score of the ERQ indicated either the presence of outliers or data skewness. Therefore, further investigation based on the method described in Hubert and Vandervieren (2008) and implemented in the R (version 4.0.4, R Core Team, 2022) package univOutl (version 0.4; D'Orazio, 2022) were conducted. In short, the interval [Q1 – 1.5*IRQ, Q3 + 1.5*IRQ] (Q1 and Q3: first and third quartile, IRQ = interquartile range) commonly used for outlier detection is adapted according to the medcouple measure for skewness. This analysis revealed two outliers at the lower tail of the distribution. In addition, these two cases revealed a mean of the reappraisal score <1.5 (range: 1–7). Given that the sample should be mentally healthy and thus, more likely to apply reappraisal, their score seems not plausible and might point to inaccuracies in filling in the ERQ. Thus, we decided to exclude these two cases from analyses. Therefore, the final sample consisted of 57 participants (*n* = 37 (64.9%) women, *n* = 20 (35.1%) men, age: *M* = 30.77 years, *SD* = 12.98 years, range = 18–59 years, *Md* = 25 years; years of education: *M* = 17.21 years, SD = 4.28 years, range = 11.50–32.00 years, *Md* = 16.00 years, further sample characteristics are shown in Table 1).

### Stimuli

The picture set for the emotion regulation paradigm comprised 16 aversive and eight neutral pictures. Aversive pictures depicted one or more people suffering and could be divided into four subcategories: homeless person, domestic violence, ill person in the hospital, and accident scene. Neutral pictures showed everyday scenes (e.g., a conversation). Pictures were taken from the International Affective Picture System (IAPS) (Lang et al., 1997) or from the Internet. The picture set was rated by an independent sample and used in previous studies (for details, see Hermann et al., 2017; Hermann et al., 2021). Pictures were rated regarding their valence and arousal on a 9-point Likert scale in an independent sample (*N* = 36; age: *M* = 26.19 years, *SD* = 4.01 years, range: 20–37 years; 41.7% women, 58.3% men). Aversive pictures were perceived as less pleasant (*M* = 2.50; *SD* = 0.84) and more arousing (*M* = 5.13; *SD* = 1.58) than neutral pictures (valence: *M* = 5.58, *SD* = 0.77; arousal: *M* = 2.00, *SD* = 0.99). Stimuli were presented on a full screen of a 27-inch monitor, which was distanced 45 inches from the participants.

### Procedure

In the experimental session, the emotion regulation paradigm was conducted. First, the procedure of the session was described, and a 5-minute baseline measurement was conducted assessing EEG, electrodermal activity, heart rate, and respiration (data will be reported elsewhere). The regulation task followed, during which participants were instructed to look at the pictures (German: ‘Betrachten’) or were explicitly told to reduce their negative feelings via distancing (German: ‘Distanzieren’) or reinterpretation (German: ‘Umdeuten’). For the ‘look’ condition, participants were told to simply watch the scene while permitting all upcoming feelings and thoughts without altering them. During distancing, participants should imagine that they were an objective/detached observer of the scene or that they do not know the person depicted on the picture. During reinterpretation, participants should imagine a concrete happy ending of the scene or that it is better than expected in order to reduce their negative feelings. Participants received a written instruction of the task and following, all strategies were trained face-to-face using a picture set independent from the one used in the regulation task. While implementing the strategy, participants should think aloud and the experimenter corrected them if the strategies were misapplied (e.g., imagining that the scene was a film clip as this imagination would not reduce negative feelings in their everyday personal live). Following this, a computer-based training of the experimental task was conducted to allow the participants to familiarize with the task. During this training session, 26 example trials were conducted with different pictures from the regulation task (8 trials/condition for aversive pictures so that each picture subcategory was presented two times; 2 trials for the look neutral condition). Afterwards, the experimenter checked the correct implementation of the tactics and clarified remaining questions.

The emotion regulation paradigm was adapted from previous studies (Hermann et al., 2017; Hermann et al., 2021), and the design was optimized for EEG data recording and analyses. It comprised two phases, which were the active emotion regulation and the re-exposure phase: The active regulation phase consisted of 96 trials with 24 trials for each of the experimental conditions (reinterpretation, distancing, look aversive, look neutral). One picture of each of the four subcategories was used for each condition showing aversive pictures. Our goal was to be closer to training and therapy effects, which is why each picture was repeated six times to enhance consolidation into memory. This is indicated by previous literature, showing that repeated reappraisal of pictures (compared with applying reappraisal only once, to watching new control images and also to having repeatedly watched pictures as many times as in the repeated reappraisal condition) leads to a lasting attenuation of the amygdala response 1 week later when the stimuli were presented again (Denny et al., 2015). These results were discussed in terms of lasting changes in the neural representation of the emotional value of the respective stimuli (Denny et al., 2015).

The assignment of specific pictures of each subcategory to conditions was randomized across participants. The emotion regulation phase consisted of six blocks, whereby each block comprised each of the four conditions (16 trials). The conditions were pseudo-randomized so that the same condition was presented no more than twice in a row. Each picture was always paired with the same regulation instruction (six times in total). Each trial started with a jittered presentation of a white fixation cross centered on a black background, followed by an instruction word for 2 s, then by the picture for 6 s, and by a subjective rating of negative feelings lasting for a maximum of 4 s or until participants pressed the middle button on the rating box (7-point Likert scale ranging from 1 = not at all to 7 = very strong). It ended with a fixation cross for 2 s. The total trial duration was 15 s.

During an approximately 30-minute break, participants completed questionnaires (e.g., movement and sports activity within the last month). They also gave post-hoc ratings for success and effort for the implementation of reinterpretation and distancing during the experimental task, respectively, as well as for the frequency and success of their daily use of both reappraisal tactics on nine-point Likert scales (results will be reported elsewhere).

Then, the re-exposure phase begun during which the 16 pictures from the active regulation phase as well as eight new pictures (4 neutral, 4 aversive) were presented, resulting in six conditions: previous look aversive, previous look neutral, previous reinterpretation, previous distancing, look new neutral, and look new aversive. Participants had not been informed that they would see the pictures from the regulation phase again and were instructed to simply attend to the pictures (they received no instruction word). The re-exposure phase consisted of 144 trials with 24 trials for each of the experimental condition and comprised six blocks (with 24 trials per block). The conditions were again pseudo-randomized so that the same condition was presented no more than twice in a row. During the re-exposure phase, stimuli were presented for 3 s each and participants again rated their negative feelings towards the stimuli (maximum of 4 s). The trials started (jittered) and ended with a white fixation cross on a black background. The total trial duration was 11 s.

Finally, participants completed a post-hoc paper-pencil awareness rating during which each picture was shown separately. They should indicate if they have seen the picture during the active regulation phase (yes or no), and if yes, with which strategy the pictures were presented before (during active regulation: look, reinterpretation, distancing, or I don’t know). In case they stated that the picture was paired with reinterpretation or distancing, participants also should indicate whether they applied this strategy again during the re-exposure phase (yes, no).

### Assessment of habitual use of cognitive reappraisal

The Emotion Regulation Questionnaire (ERQ, Gross and John, 2003; German version: Abler and Kessler, 2009) is a 10-item questionnaire capturing habitual use of cognitive reappraisal (6 items) and expressive suppression (4 items) of which only the reappraisal scale was used in our study. This subscale measures how emotions are habitually controlled by cognitively changing the meaning of a stimulus or situation so that the emotional impact is altered (e.g., “I control my emotions by changing the way I think about the situation I’m in.”). Items are rated on a 7-point Likert scale from 1 (strongly disagree) to 7 (strongly agree). It demonstrated good psychometric properties (Gross and John, 2003). The reappraisal scale showed an internal consistency of Cronbach’s *α* = 0.78 in the current study.

### Data recording and analyses

Continuous EEG was recorded from 32 Ag/AgCl scalp electrodes based on the 10/20 system (ANT Neuro, Hengelo, Netherlands), as well as two electrodes placed on the left and right mastoids. During recording, the CPz electrode served as online reference and AFz as ground electrode. Additionally, there was one facial electrode, which recorded the electro-oculogram (EOG) and was placed below the left eye. The recordings were sampled at 512 Hz and digitized with eego™ software (Version 1.8.2, eemagine Medical Imaging Solutions GmbH, Berlin, Germany). Impedance levels at all channels were kept below 5 kΩ. Offline, pre-processing was completed by using BrainVision Analyzer (BrainProducts, Gilching, Germany, Version 2.2.0.7383). EEG data were high-pass filtered at 0.02 Hz (order 2) and low-pass filtered at 30 Hz (order 4). For eye movement correction, an independent component analysis (ICA) was computed and EOG relevant ICs were identified by visual inspection and comparison to the EOG channel. Afterwards, a semiautomatic data inspection was conducted, frames were marked as bad if the voltage step was >50 μV/ms, if the maximal allowed difference of values in intervals was >200 μV, or if the activity was <0.5 μV. The whole data set as well as marked intervals were manually checked and discarded if necessary. Data of each participant were only further analyzed if at least 12 remaining artifact-free trials per condition and phase (Moran et al., 2013) were available. Based on this criterion, data from one participant were excluded from analyses regarding the active emotion regulation phase and data from another participant regarding the re-exposure phase. The number of discarded trials did not differ between conditions for the active regulation phase (*F*(3, 165) = 0.026, *p* = 0.994), nor for the re-exposure phase (*F*(3, 165) = 1.00, *p* = 0.396). Then, the data were re-referenced to the average of the left and right mastoids and segmented into epochs beginning 200 ms before picture onset to 3,000 ms following picture presentation (and from 3,000 to 6,000 ms for the active emotion regulation phase for exploratory post-hoc analyses). Segments were baseline corrected using the average activity in the 200-ms time window preceding the picture onset. Separate averages were computed for each condition (active regulation phase: look aversive, look neutral, reinterpretation, distancing; re-exposure phase: previous look aversive, previous look neutral, previous reinterpretation, previous distancing, new look aversive, new look neutral). Magnitudes of the ERP components were exported via average amplitudes for time windows 300–500 ms (P300), 500–800 ms (early LPP), 800–1,400 ms (mid LPP) and 1,400–3,000 ms (late LPP) according to previous literature (DeCicco et al., 2012; Krompinger et al., 2008; Liu et al., 2019). As a few studies point to regulation effects extending the 3,000-ms time window (Qi et al., 2017; Thiruchselvam et al., 2011), we also run exploratory post-hoc analyses for the remaining 3,000–6,000-ms time window in the active regulation phase (Tables 2 and 3). Based on literature (Hua et al., 2015; Moser et al., 2014; Parvaz et al., 2012; Paul et al., 2013) and visual inspection, we quantified the LPP as the average signal amplitude collapsed across 5 electrodes within the posterior-parietal region (CPz, Pz, POz, O1, O2).

### Statistical analyses

In their study, Qi et al. (2017) reported regulatory effects sizes of *d* = 0.40 or lager. Accordingly, a power analysis using G*Power software (Faul et al., 2007, 2009) revealed that a sample of at least 52 participants is required to detect an effect of *d* = 0.40 in a *t*-test for a dependent sample (two-tailed) with 80% power and an error probability of *α* = 0.05.

All statistical analyses were conducted with IBM SPSS Statistics (version 28). Repeated-measures analysis of variance (ANOVA) with condition as a within-factor and the reappraisal score of the ERQ as a covariate (mean centered) were conducted separately for the (lasting) effects of emotional reactivity (conditions: (previous) look aversive, (previous) look neutral) and explicit regulation (conditions: (previous) look aversive, (previous) reinterpretation, (previous) distancing). All ANOVA results were Greenhouse-Geisser corrected if the assumption of sphericity was violated. A priori planned comparisons were performed via *t*-tests for dependent samples, and *p*-values were Bonferroni-Holm-corrected (bhc), i.e., (previous) look aversive vs. (previous) reinterpretation, (previous) look aversive vs. (previous) distancing, and (previous) distancing vs. (previous) reinterpretation. Conditions regarding new pictures within the re-exposure phase (i.e., look new neutral, look new aversive) were not analyzed for the purposes of this study. Effect sizes were presented by using partial eta-squared (*η*_*p*_^2^) for *F*-tests and Cohen’s *d* for *t*-tests. When the interaction between condition and the reappraisal score of the ERQ was significant, correlational analyses followed using the difference scores (i.e., (lasting) emotional reactivity: (previous) look aversive minus (previous) look neutral; (lasting) explicit regulation: (previous) look aversive minus (previous) reinterpretation, (previous) look aversive minus (previous) distancing). All results are reported two-tailed at a significance level of *α* = 0.05 and at a trend level of *α* = 0.10.

## Results

### Main results

#### Active emotion regulation phase

- *Subjective rating of negative feelings*

##### Emotional reactivity

A significant main effect of condition for self-reported negative feelings was observed (*F*(1, 54) = 291.11, *p* < 0.001, *η*_*p*_^*2*^ = 0.844). The effect was not moderated by the habitual use of reappraisal (reappraisal score of the ERQ; see Table 2). The a priori planned *t*-test revealed that looking at negative pictures led to a higher experience of negative feelings (*M* = 4.74, *SD* = 1.58) compared with looking at neutral pictures (*M* = 1.16, *SD* = 0.34), *t* = 17.21, *p* < 0.001, *d* = 2.30.

##### Explicit emotion regulation

**Fig. 1:**
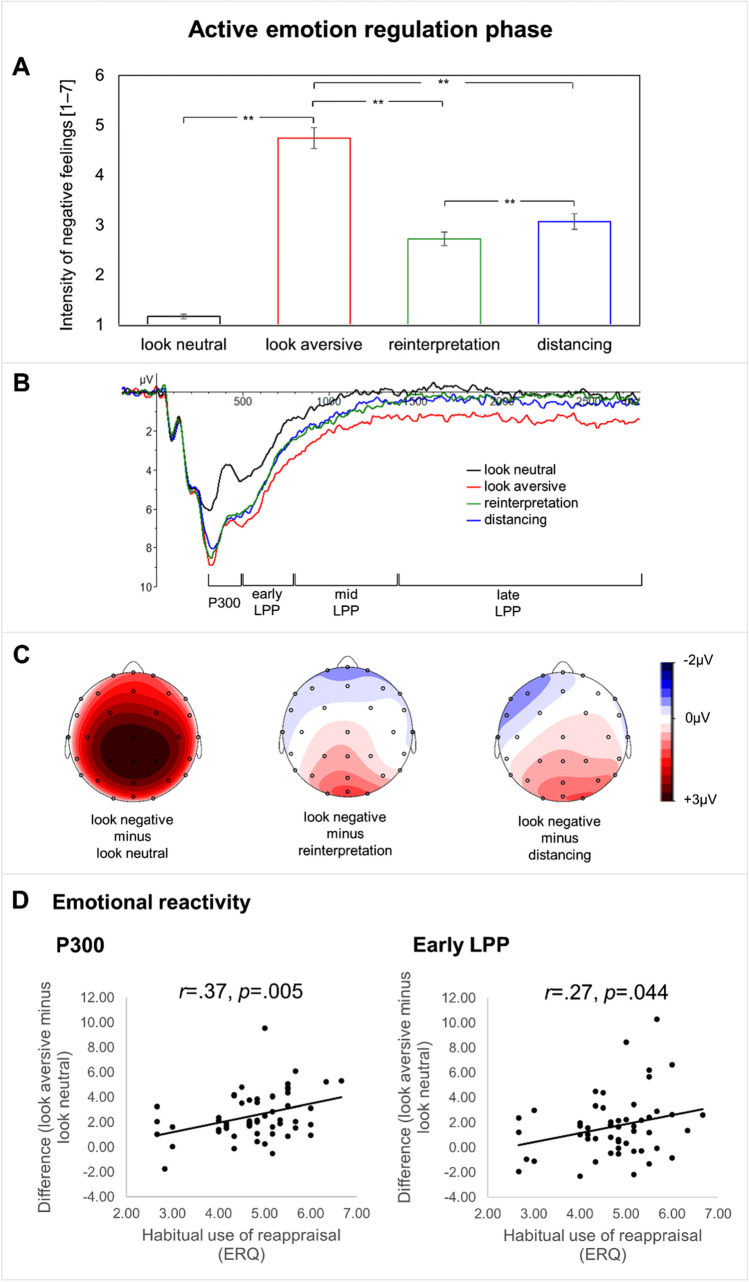
**A**. Means of self-reported negative feelings during the active emotion regulation phase for each condition. Error bars depict standard errors of the means. **B.** Event-related potentials following picture presentation pooled at posterior-parietal sides (CPz, Pz, OPz, O1, O2) during the active emotion regulation phase. **C.** Topographical distribution of respective difference waves during 300–3,000 ms. **D.** Association between habitual reappraisal (measured by the ERQ) and the difference score of emotional reactivity (look aversive minus look neutral). **p < 0.001

A significant main effect of condition was given (*F*(1.40, 75.66) = 91.93, *p* < 0.001, *η*_*p*_^*2*^ = 0.630), which was not moderated by the habitual use of reappraisal (see Table 3). Planned *t*-tests showed that both cognitive reappraisal tactics significantly reduced negative feelings compared with passively looking at them (look aversive (*M* = 4.74, *SD* = 1.58) vs. reinterpretation (*M* = 2.72, *SD* = 1.01): *t* = 11.11, *p* < 0.001, *d* = 1.48; look aversive (*M* = 4.74, *SD* = 1.58) vs. distancing (*M* = 3.07, *SD* = 1.18): *t* = 9.18, *p* < 0.001, *d* = 1.23; Fig. 1A). Reinterpretation resulted in a significant stronger decrease of negative feelings compared with distancing (distancing vs. reinterpretation: *t* = 3.77, *p* < 0.001, *d* = 0.50).

### ERP data

#### Emotional reactivity

ERPs of the different conditions and difference maps are shown in Fig. 1B and C. Mean amplitudes at posterior-parietal sides revealed a significant main effect of condition for the P300 (*F*(1, 54) = 114.93, *p* < 0.001, *η*_*p*_^*2*^ = 0.680), the early LPP (*F*(1, 54) = 28.97, *p* < 0.001, *η*_*p*_^*2*^ = 0.347), the mid LPP (*F*(1, 54) = 19.258, *p* < 0.001, *η*_*p*_^*2*^ = 0.263), as well as for the late LPP (*F*(1, 54) = 8.171, *p* = 0.006, *η*_*p*_^*2*^ = 0.131). An explorative analysis demonstrated that the emotional reactivity lasted over the whole picture presentation (Table 2). The P300 and the early LPP were significantly moderated by the habitual use of reappraisal (P300: *F*(1, 54) = 8.644, *p* = 0.005, *η*_*p*_^*2*^ = 0.138; r(look aversive minus look neutral) = 0.37, *p* = 0.005; early LPP (*F*(1, 54) = 4.25, *p* = 0.044, *η*_*p*_^*2*^ = 0.073; r(look aversive minus look neutral) = 0.27, *p* = 0.044; Fig. 1D). Neither the mid, nor the late LPP showed a significant interaction effect (Table 2). A priori planned pairwise comparisons demonstrated that looking at aversive pictures elicited significantly larger ERPs than looking at neutral ones in all time windows (P300: look aversive (*M* = 7.31, *SD* = 3.46) vs. look neutral (*M* = 4.79, *SD* = 3.35), *t* = 10.05, *p* < 0.001, *d* = 2.30; early LPP: look aversive (*M* = 4.87, *SD* = 2.95) vs. look neutral (*M* = 3.17, *SD* = 2.70), *t* = 5.23, *p* < 0.001, *d* = 0.70; mid LPP: look aversive (*M* = 1.90, *SD* = 2.72) vs. look neutral (*M* = 0.56, *SD* = 2.54), *t* = 4.40, *p* < 0.001, *d* = 0.59; late LPP: look aversive (*M* = 1.38, *SD* = 3.74) vs. look neutral (*M* = 0.44, *SD* = 3.18), *t* = 2.85, *p* = 0.006, *d* = 0.38; and 3,000–6,000-ms time window; Table 2).

#### Explicit emotion regulation

The amplitudes in the P3 did not significantly differ between the conditions look aversive, reinterpretation and distancing, and there was no moderation effect of the habitual use of reappraisal (measured by the ERQ; Table 3). However, a main effect of condition was given during the early LPP (*F*(2,108) = 3.318, *p* = 0.040, *η*_*p*_^2^ = 0.058), the mid LPP (*F*(2,108) = 3.683, *p* = 0.028, *η*_*p*_^2^ = 0.064), as well as the late LPP (*F*(2,108) = 4.053, *p* = 0.020, *η*_*p*_^2^ = 0.070). This effect also was evident at trend level during the 3,000 to 6,000-ms time window (Table 3). Habitual use of reappraisal did not moderate the regulatory effect in the inspected time windows. Planned pairwise comparisons between looking at aversive pictures and reinterpretation showed that the implementation of reinterpretation resulted in decreases of the LPP amplitudes in the early (trend), middle (trend), and late time window (early LPP: look aversive (*M* = 4.87, *SD* = 2.95) vs. reinterpretation (*M* = 4.18, *SD* = 2.81), *t* = 2.28, *p* = 0.081, bhc, *d* = 0.304), mid LPP: look aversive (*M* = 1.90, *SD* = 2.72) vs. reinterpretation (*M* = 1.25, *SD* = 2.85), *t* = 2.22, *p* = 0.062, bhc, *d* = 0.297, late LPP: look aversive (*M* = 1.38, *SD* = 3.74) vs. reinterpretation (*M* = 0.40, *SD* = 3.66), *t* = 2.57, *p* = 0.039, bhc, *d* = 0.343). After controlling for multiple comparisons, post-hoc *t*-tests did not indicate that the effect of reinterpretation lasted over the 3,000 to 6,000-ms time window.

Distancing did not significantly differ from looking at aversive pictures for the P300, for the early LPP and for the 3,000 to 6,000-ms time window (Table 3). However, distancing reduced the LPP compared to looking at aversive pictures in the mid and late LPP on trend level (mid LPP: look aversive (*M* = 1.90, *SD* = 2.72) vs. distancing (*M* = 1.00, *SD* = 2.97), *t* = 2.33, *p* = 0.072, bhc, *d* = 0.311; late LPP: look aversive (*M* = 1.38, *SD* = 3.74) vs. distancing (*M* = 0.45, *SD* = 3.23), *t* = 2.16, *p* = 0.070, bhc, *d* = 0.285).

Distancing and reinterpretation did not significantly differ from each other neither for the P300, the early LPP, the mid LPP, the late LPP, nor for the 3,000 to 6,000-ms time window (all *p* > 0.467; Table 3).

#### Re-exposure phase

- *Subjective rating of negative feelings*

##### Emotional reactivity

**Fig. 2:**
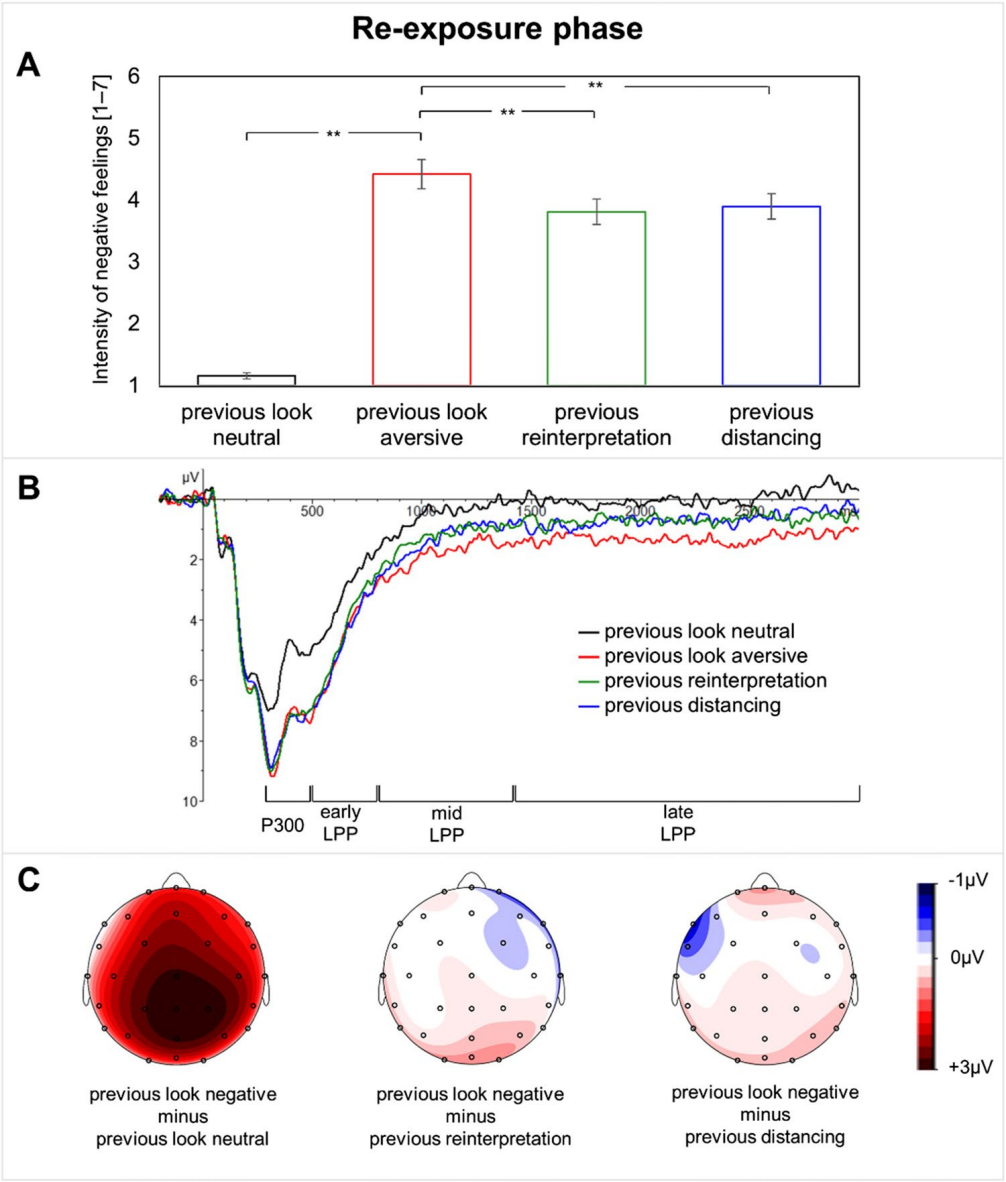
**A**. Means of self-reported negative feelings during the re-exposure phase for each condition. Error bars depict standard errors of the means. **B**. Event-related potentials following picture presentation pooled at posterior-parietal sides (CPz, Pz, OPz, O1, O2) during the re-exposure phase. **C**. Topographical distribution of respective difference waves during 300–3,000 ms. **p < 0.001

A significant main effect of condition was detected for self-reported negative feelings (*F*(1, 54) = 191.05, *p* < 0.001, *η*^*2*^ = 0.78), which was not moderated by the habitual use of reappraisal (measured with the ERQ; Table 4). The a priori planned *t*-test indicated that looking again at aversive pictures led to increased negative feelings (*M* = 4.42, *SD* = 1.77) compared with looking again at neutral pictures (previous look aversive vs. previous look neutral (*M* = 1.16, *SD* = 0.37): *t* = 13.91, *p* < 0.001, *d* = 1.86; Fig. 2A).

##### Explicit emotion regulation

The ANOVA revealed a significant main effect of condition (*F*(1.70, 91.75) = 40.43, *p* < 0.001, *η*^*2*^ = 0.43), whereby the interaction between condition and the habitual use of reappraisal was not significant (Table 5). Planned post-hoc *t*-tests showed that both tactics had a lasting regulation effect, as indicated by reduced negative feelings for each tactic compared with previous looking at aversive pictures (previous look aversive (*M* = 4.42, *SD* = 1.77) vs. previous reinterpretation (*M* = 3.81, *SD* = 1.54): *t* = 7.88, *p* < 0.001, *d* = 1.05; previous look aversive (*M* = 4.42, *SD* = 1.77) vs. previous distancing (*M* = 3.90, *SD* = 1.55): *t* = 6.37, *p* < 0.001, *d* = 0.85; Fig. 2A). Negative feelings did not significantly differ between previous distancing and previous reinterpretation (*t* = 1.53, *p* = 0.132, *d* = 0.21).

- *ERP Data*

##### Emotional reactivity

ERPs of the different conditions and difference maps are shown in Fig. 2B and C. A significant main effect of condition was observed for the P300 (*F*(1, 54) = 39.23, *p* < 0.001, *η*^*2*^ = 0.42), the early LPP (*F*(1, 54) = 16.44, *p* < 0.001, *η*^*2*^ = 0.23), the mid LPP (*F*(1, 54) = 10.83, *p* = 0.002, *η*^*2*^ = 0.17), and the late LPP (*F*(1, 54) = 8.67, *p* = 0.005, *η*^*2*^ = 0.14). The interaction between condition and the reappraisal score of the ERQ was not significant (Table 4). A priori planned pairwise comparisons showed that looking again at aversive pictures led to significantly larger ERP amplitudes than looking again at neutral ones for the P300 (previous look aversive (*M* = 7.73, *SD* = 3.82) vs. previous look neutral (*M* = 5.61, *SD* = 4.07): *t* = 6.32, *p* < 0.001, *d* = 0.84), the early LPP (previous look aversive (*M* = 4.79, *SD* = 2.95) vs. previous look neutral (*M* = 3.34, *SD* = 2.85): *t* = 4.09, *p* < 0.001, *d* = 0.55), the mid LPP (previous look aversive (*M* = 2.04, *SD* = 2.71) vs. previous look neutral (*M* = 0.67, *SD* = 2.64): *t* = 3.32, *p* < 0.001, *d* = 0.44), and the late LPP (previous look aversive (*M* = 1.55, *SD* = 2.95) vs. previous look neutral (*M* = 0.20, *SD* = 2.70): *t* = 2.97, *p* = 0.004, *d* = 0.40).

##### Explicit emotion regulation

The ANOVA did not show a main effect of condition, demonstrating that there were no significant differences in the ERP amplitudes over the inspected time windows between previous looking at aversive pictures, previous reinterpretation and previous distancing. Furthermore, no moderation effect was given. All results are reported in Table 5.

### Further Analyses

#### Post-hoc success and effort ratings of reappraisal tactics

Participants rated to be more successful in reinterpretation (*M* = 6.42, *SD* = 1.64) compared with distancing (*M* = 5.44, *SD* = 2.36), *t*(54) = 2.78, *p* = 0.008, *d* = 0.37) regarding the active regulation phase. There was no difference in effort ratings for the implementation of both tactics (reinterpretation: *M* = 4.82, *SD* = 2.15, distancing: *M* = 5.25, *SD* = 2.41, *t* = −1.11, *p* = 0.272, *d* = −0.15).

#### Post-hoc awareness ratings

Participants reported to remember having seen pictures with a reinterpretation history more often compared to look aversive (*t* = 2.07, *p* = 0.044, *d* = 0.31), but not compared to distancing (*t* = 0.40, *p* = 0.688, *d* = 0.06). Distancing and look aversive did not differ from each other (*t* = 1.65, *p* = 0.105, *d* = 0.25). Participants further correctly remembered the instruction more often for reinterpretation compared to look aversive (*t* = 4.41, *p* < 0.001, *d* = 0.58) and compared with distancing (*t* = 3.83, *p* < 0.001, *d* = 0.51). However, there was no difference in correctly remembering the instruction for distancing and look aversive (*t* = 1.76, *p* = 0.084, *d* = 0.23). If they correctly identified the instruction, they also applied the tactic during re-exposure more often (without being instructed to) for reinterpretation than for distancing (*t* = 9.31, *p* < 0.001, *d* = 1.28).

## Discussion

This is the first ERP study investigating immediate and lasting effects of emotional reactivity as well as reinterpretation and distancing in association with the habitual use of cognitive reappraisal. Aversive pictures provoked stronger negative feelings and a higher amplitude in ERPs compared with the presentation of neutral pictures during both, the active regulation and the re-exposure phase. A higher habitual use of reappraisal was associated with stronger emotional reactivity in the P300 and early LPP during the active regulation phase. The implementation of both tactics led to reduced negative feelings, while reinterpretation reduced negative feelings to a higher extent compared with distancing. Reinterpretation attenuated the early, mid (both at trend level) and late LPP, distancing the mid and late LPP (both at trend level). For lasting effects, looking at previously reappraised pictures (reinterpretation and distancing) led to less negative feelings compared to looking again at aversive pictures. However, lasting regulatory effects were neither found for reinterpretation nor for distancing on the electrocortical level.

In line with a large body of research, looking at aversive compared with neutral pictures led to a stronger experience of negative feelings (Hermann et al., 2021) and ERP amplitudes (P3, LPP) during the whole presentation time of the pictures (Hajcak et al., 2010; Hajcak and Nieuwenhuis, 2006; Hermann et al., 2021; Paul et al., 2013; Qi et al., 2017; Thiruchselvam et al., 2011). On the electrocortical level, this differential effect has further shown to be stable over picture repetitions up to 60 times (Codispoti et al., 2006) as well as over time, when pictures were shown again one day later (Ferrari et al., 2020).

Moreover, both tactics successfully lowered negative feelings during active emotion regulation which also is in line with previous research (Hermann et al., 2021; Shiota and Levenson, 2012; Webb et al., 2012), with reinterpretation being more effective than distancing. Furthermore, participants rated to be more successful in reinterpretation than in distancing whereby both tactics did not differ in effort ratings. This differential effect on negative feelings might result from reinterpretation leading to more positive feelings (“imagining a happy ending”) and distancing to a more neutral response (Hermann et al., 2021; Qi et al., 2017; Shiota and Levenson, 2012). This also corresponds with results of a previous study showing distancing to lead to a stronger reduction in arousal ratings and reinterpretation to a higher increase in valence ratings (both compared with each other; Qi et al., 2017). Furthermore, this possible differential effect of reinterpretation on valence might explain differences in the success ratings, showing that participants indicated to be more successful in downregulating their emotions via reinterpretation compared with distancing.

On the electrocortical level, both tactics attenuated the LPP compared to passively looking at aversive pictures in the early (reinterpretation only), as well as in the middle time window (at trend level) and lasted over the late time window (for distancing on trend level only, both compared to looking at aversive pictures). In contrast to a previous study by Qi et al. (2017), our results did not show a stronger nor an earlier reduction of the LPP for distancing compared with reinterpretation. This missing differential effect might be due to methodological differences between both studies, e.g., repeated picture presentation in our study, possibly leading to a stronger consolidation and faster recall of the altered memory representation. As a consequence, this might cancel out effectiveness and timing differences between both tactics in the LPP. In sum, both tactics were effective in reducing negative feelings and LPP amplitudes during the active regulation phase. Stronger effects for reinterpretation were only evident on the self-report level, indicating different underlying mechanisms which were not reflected in the ERPs.

For lasting effects, we found that the implementation of both tactics led to reduced negative feelings when the pictures were presented again half an hour later. This is in line with a previous study (Hermann et al., 2021) that used the same stimulus material and demonstrated lasting effects on negative feelings even 1 week later. Differential effects of both tactics were only observed in the awareness ratings, revealing that participants recognized and correctly remembered the instruction more frequently for reinterpreted pictures than pictures passively looked at or distanced from in the first phase. This higher awareness for reinterpretation corresponds with studies showing improved memory effects for reinterpreted pictures (Willroth and Hilimire, 2016), possibly reflecting a deeper elaboration of the picture content for reinterpretation in the current study.

On the electrocortical level, we did not find substantially lasting effects of reinterpretation or distancing in the LPP. Qi et al. (2017) also found no lasting effect for reinterpretation but for distancing. Differences in the results, especially our missing regulatory findings, might come from repeated picture presentations, possibly leading to a weakening of the LPP amplitudes in the look aversive condition in the current study. In line with this assumption, attending to a picture three times led to a decrease in the LPP in a previous study (Paul et al., 2013). Therefore, as the pictures in our study were paired six times with the same instruction during active regulation, this might have reduced arousal in general upon re-exposure. Another possible explanation for the missing LPP effect of reinterpretation might stem from its impact on positive emotions, as discussed above. A positive reinterpretation of the depicted scenes might lead to enhanced arousal (and associated increased LPP amplitudes) during re-exposure, which might be comparable to the arousal evoked from looking again at aversive pictures repeatedly watched in the first phase.

While a more frequent habitual use of reappraisal has shown to be related to a decreased LPP during passively watching negative pictures (Harrison and Chassy, 2019), we found, unlike expected, a higher habitual use of reappraisal to be associated with stronger amplitudes in the P300 and the early LPP for emotional reactivity during the active regulation phase. A heightened P300 is related to threat and self-referential processing (Wang et al., 2021), whereby attention seems to be driven automatically towards salient stimuli (Hajcak and Foti, 2020). Especially the later portion of the P300 might be produced by memory updating operations necessary for top-down control of emotions (Polich, 2007). Therefore, an increased reactivity towards aversive stimuli might reflect a heightened preparedness for regulation and the initiation of automatic control mechanisms in response to salient stimuli. We found no moderation by the habitual use of reappraisal for distancing or reinterpretation, which has been reported for reinterpretation only in a previous study (Moser et al., 2014). Individuals might only differ during spontaneous responding to emotional stimuli but are capable of down-regulating negative feelings when instructed to do so. Upon re-exposure, the repeated presentation of pictures might have reduced arousal over all conditions, which is why a moderating role of habitual use of reappraisal might be missing for emotional reactivity as well as for distancing and reinterpretation.

Our study also has some limitations: Previous research suggests that different ethnic groups differ in their expression of emotions and in emotion regulation (Consedine and Magai, 2002; Weiss et al., 2022). Unfortunately, we did not collect information about ethnicity and race. Based on the composition of the population in which the study was conducted, it can be assumed that participants were predominantly of German ethnicity and Caucasian, which is why our results are limited to this sample population. Furthermore, given that the lifetime prevalence for mental disorders is high (Kessler et al., 2007), it should be noted that our sample might have been healthier than the average population, as only participants were included who have never fulfilled the criteria for any mental disorder. However, as there exist differences in emotional reactivity (Granros et al., 2022; MacNamara et al., 2016) and regulation (Paul et al., 2016) between healthy and clinical populations, studying healthy individuals is essential to unravel observed effects. Because we did not assess valence and arousal, but negative feelings as an outcome of reappraisal, it is not possible to disentangle differential effects of reinterpretation and distancing on these dimensions. We cannot fully answer our above-mentioned questions about reinterpretation leading to an increase of positive feelings. Differences in the effectiveness of reinterpretation vs. distancing might be traced to how aversive the stimulus material is perceived by the participants. According to research in emotion regulation choice (Sheppes and Levin, 2013), individuals prefer engaging regulation strategies (such as reinterpretation) more often when the picture material is less aversive. This might be the case for our study, as we used four specific subcategories of aversive pictures and further repeatedly presented our pictures. Therefore, participants might have experienced the pictures as less aversive compared with other studies where more heterogeneous pictures were presented only once (Thiruchselvam et al., 2011). When lasting effects of explicit emotion regulation are investigated, it should be noted that a demand to regulate is given in the active regulation phase but not in the re-exposure phase, which might differentially influence the results in both phases. This might especially increase the immediate effects in the rating of negative feelings. Furthermore, on a neural level, explicit compared with implicit emotion regulation has been shown to involve common as well as different brain activation patterns (Braunstein et al., 2017), which might contribute in part to a lack of lasting ERP findings in our study. However, studies that showed pictures only once in the active emotion-regulation phase did find lasting regulatory effects on ERPs (Qi et al., 2017; Thiruchselvam et al., 2011), favoring the assumption that the missing effects in our study might be traced to repeated picture presentation as illustrated above.

Moreover, the current and previous studies used different instruction practices. In our study, e.g., participants were corrected if they imagined something that is not possible in real life. Other studies (Thiruchselvam et al., 2011) explicitly suggest individuals to imagine that the depicted scene is not real. Studies also differ in the inspected electrode or cluster as well as the observed time windows when investigating emotion-regulatory effects in ERPs making direct comparisons between findings more difficult.

In summary, our findings demonstrate the immediate effects of reinterpretation and distancing on negative feelings and the LPP. Lasting effects were only evident on a subjective level but not found in the EEG, which might result from repeated picture presentation, leading to weakened general arousal in response to the stimuli. Moreover, a higher use of habitual reappraisal was associated with stronger emotional reactivity indexed by higher P3 and LPP amplitudes for passively viewing aversive compared with neutral pictures and might be interpreted in terms of a higher preparedness to regulate. However, there was no evidence for substantial differences between both reappraisal tactics, especially regarding the LPP, probably indicating that the LPP does not fully capture underlying differential mechanisms (e.g., effects on valence). The findings of the current study might contribute to a deeper understanding of immediate and lasting effects of different emotion regulation tactics. Moreover, our results regarding the association with individual differences in the habitual use of cognitive reappraisal might be especially important for improving psychotherapeutic treatments.

## Data Availability

All data are available upon request to the corresponding author. The experiment was not preregistered.

## Appendix

**Table 1:** Sample characteristics

	n = 57
Gender *N* (women / men / nonbinary)	37 / 20 / 0
Age (years) *M* (*SD*), range	30.77 (12.98),18.00–59.00
Years of education *M* (*SD*), range	17.21 (4.28), 11.50–32.00
Highest level of education, *N*	
University degree or similar	17
German Abitur or similar^1^	38
Secondary school diploma	2
Employment status^2^, *N*	
Currently employed	56
Other	1
Relationship status, *N*	
Single	20
Solid Partnership	25
Married	8
Married, living separately	3
Divorced	1

^1^This is approximately equivalent to an A-level degree.

^2^Further answer options were: Sick leave (*n* = 0), disability (*n* = 0), or old age pension (*n* = 0). The one participant indicating “Other” was on parental leave at that time

**Table 2:** Results of emotional reactivity during the active emotion regulation phase

	ANOVA (within-subject factor: condition; covariate: reappraisal score of ERQ)		Look negative *M* (*SD*)	Look neutral *M* (*SD*)	Planned comparison *t*-test look negative vs. look neutral
	Main effect of condition	Interaction condition * reappraisal score (ERQ)			
Rating	*F*(1, 54) = 291.11, *p* < 0.001, *η*_*p*_^2^ = 0.84	*F*(1, 54) = 0.07, *p* = 0.787, *η*_*p*_^2^ = 0.001	4.74 (1.58)	1.16 (0.34)	*t =* 17.21, *p* < 0.001, *d =* 2.30
P3 (300 – 500 ms)	*F*(1, 54) = 114.93, *p* < 0.001, *η*_*p*_^2^ = 0.68	*F*(1, 54) = 8.64, *p* = 0.005, *η*_*p*_^2^ = 0.14	7.31 (3.46)	4.79 (3.35)	*t =* 10.05, *p* < 0.001, *d =* 1.34
early LPP (500–800 ms)	*F*(1, 54) = 28.97, *p* < 0.001, *η*_*p*_^2^ = 0.35	*F*(1, 54) = 4.25, *p* = 0.044, *η*_*p*_^2^ = 0.07	4.87 (2.95)	3.17 (2.70)	*t =* 5.23, *p* < 0.001, *d =* 0.70
mid LPP (800–1400 ms)	*F*(1, 54) = 19.26, *p* < 0.001, *η*_*p*_^2^ = 0.26	*F*(1, 54) = 0.81, *p* = 0.374, *η*_*p*_^2^ = 0.02	1.90 (2.72)	0.56 (2.54)	*t =* 4.40, *p* < 0.001, *d =* 0.59
late LPP (1400–3000 ms)	*F*(1, 54) = 8.17, *p* = 0.006, *η*_*p*_^2^ = 0.13	*F*(1, 54) = 1.53, *p* = 0.222, *η*_*p*_^2^ = 0.03	1.38 (3.74)	0.44 (3.18)	*t =* 2.85, *p* = 0.006, *d =* 0.38
3000–6000 ms	*F*(1, 54) = 6.48, *p* = 0.014, *η*_*p*_^2^ = 0.11	*F*(1, 54) = 2.04, *p* = 0.159, *η*_*p*_^2^ = 0.04	1.97 (6.33)	1.08 (5.18)	*t =* 2.52, *p* = 0.015, *d =* 0.34

Planned post-hoc *t*-tests were only conducted, when indicated by a significant/trend level main effect in the ANOVA. ERQ = Emotion regulation questionnaire (Gross & John, 2003)

**Table 3:** Results of explicit emotion regulation during the active emotion regulation phase

	ANOVA (within-subject factor: condition; covariate: reappraisal score of ERQ)		look negative *M* (*SD*)	re-interpretation M (*SD*)	dis-tancing M (*SD*)	planned comparisons *t*-tests		
	main effect of condition	interaction condition * reappraisal score (ERQ)				look negative vs. reinter-pretation	look negative vs. distancing	distancing vs. reinter-pretation
Rating	*F*(1.40, 75.66) = 91.93, *p* < 0.001, *η*_*p*_^2^ = 0.63	*F*(1.40, 75.66) = 0.05, *p* = 0.894, *η*_*p*_^2^ = 0.001	4.74 (1.58)	2.72 (1.01)	3.07 (1.18)	*t =* 11.11, *p* < 0.001, *d =* 1.49	*t =* 9.18, *p* < 0.001, *d =* 1.23	*t =* 3.77, *p* < 0.001, *d =* 0.50
P3 (300–500 ms)	*F*(2,108) = 1.08, *p* = 0.344, *η*_*p*_^2^ = 0.02	*F*(2,108) = 1.08, *p* = 0.344, *η*_*p*_^2^ = 0.02	7.31 (3.46)	7.02 (3.67)	6.95 (3.99)			
early LPP (500–800 ms)	*F*(2,108) = 3.32, *p* = 0.040, *η*_*p*_^2^ = 0.06	*F*(2,108) = 2.51, *p* = 0.086, *η*_*p*_^2^ = 0.04	4.87 (2.95)	4.18 (2.81)	4.19 (3.20)	*t =* 2.28, *p* = 0.081, *d =* 0.30	*t =* 1.96, *p* = 0.110, *d =* 0.26	*t =* 0.03, *p* = 0.974, *d =* 0.004
mid LPP (800–1400 ms)	*F*(2,108) = 3.68, *p* = 0.028, *η*_*p*_^2^ = 0.06	*F*(2,108) = 0.87, *p* = 0.421, *η*_*p*_^2^ = 0.02	1.90 (2.72)	1.25 (2.85)	1.00 (2.97)	*t* = 2.22, *p* = 0.062, *d =* 0.30	*t* = 2.33, *p* = 0.072, *d =* 0.31	*t* = −0.73, *p* = 0.467, d = −0.01
late LPP (1400–3000 ms)	*F*(2,108) = 4.05, *p* = 0.020, *η*_*p*_^2^ = 0.07	*F*(2,108) = 0.81, *p* = 0.447, *η*_*p*_^2^ = 0.02	1.38 (3.74)	0.40 (3.66)	0.45 (3.23)	*t* = 2.57, *p* = 0.039, *d =* 0.34	*t =* 2.16, *p* = 0.070, *d =* 0.29	*t =* 0.13, *p* = 0.894, *d =* 0.02
3000 – 6000ms	*F*(2,108) = 2.88, *p* = 0.060, *η*_*p*_^2^ = 0.05	*F*(2,108) = 0.99, *p* = 0.375, *η*_*p*_^2^ = 0.02	1.97 (6.33)	1.05 (6.23)	1.18 (5.65)	*t =* 2.17, *p* = 0.102, *d =* 0.29	*t =* 1.86, *p* = 0.136, *d =* 0.25	t = 0.33, *p* = 0.742, d = 0.04

Planned post-hoc *t*-tests were only conducted, when indicated by a significant/trend level main effect in the ANOVA and *p*-values are Bonferroni-Holm corrected. ERQ = Emotion regulation questionnaire (Gross and John, 2003)

**Table 4:** Results of emotional reactivity during the re-exposure phase

	ANOVA (within-subject factor: condition; covariate: reappraisal score of ERQ)		Previous look negative *M* (*SD*)	Previous look neutral *M* (*SD*)	Planned comparison *t*-test previous look negative vs. previous look neutral
	Main effect of condition	Interaction condition * reappraisal score (ERQ)			
Rating	*F*(1, 54) = 191.05, *p < 0.*001, *η*_*p*_^2^ = 0.78	*F*(1, 54) = 0.30, *p* = 0.587, *η*_*p*_^2^ = 0.01	4.42 (1.77)	1.16 (0.37)	*t* = 13.91, *p* < 0.001, *d =* 1.86
P3 (300–500 ms)	*F*(1, 54) = 39.23, *p < 0.*001, *η*_*p*_^2^ = 0.42	*F*(1, 54) = 0.10, *p* = 0.754, *η*_*p*_^2^ = 0.002	7.73 (3.82)	5.61 (4.07)	*t* = 6.32, *p* < 0.001, *d =* 0.84
Early LPP (500–800 ms)	*F*(1, 54) = 16.44, *p < 0.*001, *η*_*p*_^2^ = 0.23	*F*(1, 54) = 0.04, *p* = 0.843, *η*_*p*_^2^ = 0.001	4.79 (2.95)	3.34 (2.85)	*t* = 4.09, *p* < 0.001, *d =* 0.55
Mid LPP (800–1400 ms)	*F*(1, 54) = 10.83, *p* = 0.002, *η*_*p*_^2^ = 0.17	*F*(1, 54) = 0.17, *p* = 0.684, *η*_*p*_^2^ = 0.003	2.04 (2.71)	0.67 (2.64)	*t* = 3.32, *p* < 0.001, *d =* 0.44
Late LPP (1400–3000 ms)	*F*(1, 54) = 8.67, *p* = 0.005, *η*_*p*_^2^ = 0.14	*F*(1, 54) = 0.07, *p* = 0.795, *η*_*p*_^2^ = 0.001	1.55 (2.95)	0.20 (2.70)	*t* = 2.97, *p* = 0.004, *d =* 0.40

Planned post hoc *t*-tests were only conducted, when indicated by a significant/trend level main effect in the ANOVA. ERQ = Emotion regulation questionnaire (Gross and John, 2003)

**Table 5:** Results of explicit emotion regulation during the re-exposure phase

	ANOVA (within-subject factor: condition; covariate: reappraisal score of ERQ)		Previous look negative *M* (SD)	Previous re-interpretation M (SD)	Previous dis-tancing M (SD)	Planned comparisons *t*-tests		
	Main effect of condition	Interaction condition * reappraisal score (ERQ)				Previous look negative vs. previous reinter-pretation	Previous look negative vs. previous distancing	Previous distancing vs. previous reinter-pretation
Rating	*F*(1.70, 91.75) = 40.43, *p* < 0.001, *η*_*p*_^2^ = 0.43	*F*(1.70, 91.75) = 0.001, *p* = 0.997, *η*_*p*_^2^ < 0.001	4.42 (1.77)	3.81 (1.54)	3.90 (1.55)	*t =* 7.88, *p* < 0.001, *d =* 1.05	*t =* 6.37, *p* < 0.001, *d =* 0.85	*t =* 1.53, *p* = 0.132, *d =* 0.21
P3 (300–500 ms)	*F*(2,108) = 0.04, *p* = 0.960, *η*_*p*_^2^ = 0.001	*F*(2,108) = 2.02, *p* = 0.137, *η*_*p*_^2^ = 0.04	7.73 (3.82)	7.70 (4.14)	7.64 (4.36)			
early LPP (500–800 ms)	*F*(1.77,95.58) = 0.20, *p* = 0.790, *η*_*p*_^2^ = 0.004	*F*(1.77,95.58) = 2.13, *p* = 0.130, *η*_*p*_^2^ = 0.04	4.79 (2.95)	4.54 (3.13)	4.71 (3.23)			
mid LPP (800–1400 ms)	*F*(1.66, 89.54) = 1.32, *p* = 0.269, *η*_*p*_^2^ = 0.024	*F*(1.66, 89.54) = 0.915, *p* = 0.388, *η*_*p*_^2^ = 0.02	2.04 (2.71)	1.41 (3.06)	1.44 (2.64)			
late LPP (1400–3000 ms)	*F*(2,108) = 2.03, *p* = 0.137, *η*_*p*_^2^ = 0.04	*F*(2,108) = 1.17, *p* = 0.315, *η*_*p*_^2^ = 0.02	1.55 (2.95)	0.74 (2.73)	0.74 (2.99)			

Planned post-hoc *t*-tests were only conducted, when indicated by a significant/trend level main effect in the ANOVA. ERQ = Emotion regulation questionnaire (Gross and John, 2003)
